# Antidepressant Prescribing and Suicide/Self-Harm by Young Australians: Regulatory Warnings, Contradictory Advice, and Long-Term Trends

**DOI:** 10.3389/fpsyt.2020.00478

**Published:** 2020-06-05

**Authors:** Martin Whitely, Melissa Raven, Jon Jureidini

**Affiliations:** ^1^John Curtin Institute of Public Policy, Curtin University, Perth, WA, Australia; ^2^Critical and Ethical Mental Health Research Group, Robinson Research Institute, University of Adelaide, Adelaide, SA, Australia

**Keywords:** antidepressants, suicide, self-harm, adolescents, children, US Food and Drug Administration, backlash, Australia

## Abstract

In 2004, the US Food and Drug Administration (FDA) controversially issued a black box warning that antidepressants were associated with an increased risk of suicidal thoughts and behaviours in people aged under 18 years. In 2007, the warning was expanded to include young adults aged under 25 years. In 2005, the Australian Therapeutic Goods Administration responded to the FDA warning by requiring Product and Consumer Information leaflets to be updated to reflect the risk. However, there was considerable debate, and at times emotive backlash, in academic journals and the international media. Prominent US and Australian mental health organisations and psychiatrists challenged the FDA warning. They argued that, on balance, antidepressant use was likely to reduce the risk of suicide. Several ecological studies were cited misleadingly as evidence that decreasing antidepressant use increases suicide risk. From 2008 to 2018, Australian per-capita child, adolescent and young adult antidepressant dispensing (0–27 years of age) and suicide (0–24 years) rates have increased approximately 66% and 49%, respectively. In addition, there was a 98% increase in intentional poisonings among 5 to 19 year-olds in New South Wales and Victoria between 2006 and 2016, with substantial overlap between the most commonly dispensed psychotropics and the drugs most commonly used in self-poisoning. These results do not support claims that increased antidepressant use reduces youth suicide risk. They are more consistent with the FDA warning and the hypothesis that antidepressant use increases the risk of suicide and self-harm by young people. Causal relationships cannot be established with certainty until there is a vast improvement in post-marketing surveillance. However, there is clear evidence that more young Australians are taking antidepressants, and more young Australians are killing themselves and self-harming, often by intentionally overdosing on the very substances that are supposed to help them.

## Strengths and Limitations

Strengths of the paper include:

- This review uses long-term, real-world data to analyse how competing perspectives on antidepressant use and youth suicide and self-harm fit with Australian evidence.
- Australian dispensing data are high quality.
- Australian suicide statistics are more rigorous than those of many other countries.
- This review analyses the role of influential players in the Australian mental health arena, a neglected focus of research.

Limitations of the paper include:

- Antidepressant dispensing data cover the age-range 0–27 years; however, suicide data cover ages 0–24 years.
- Antidepressant data are for financial years (July to June), but suicide data are for calendar years.
- Antidepressant dispensing data collection changed in 2011/12, including low-cost prescriptions that had previously been excluded.
- Self-harm is under-reported, because many self-harm events do not result in treatment.
- Many factors will have impacted suicide rates over the period analysed and it is impossible to isolate the effects of specific factors.

## Introduction

In 2004, the US Food and Drug Administration (FDA) issued a black box warning (the highest level of warning) that using antidepressants was associated with an increased risk of suicidal thinking and behaviour in people under 18 years of age with depression and other psychiatric disorders. In 2007, the warning was expanded to include young adults aged under 25 years (1, 2). The warning was a result of an FDA analysis of short-term trials of antidepressants in children and adolescents that showed “a relative risk for suicidal behaviour or ideation of 1.95 (95% confidence interval 1.28 to 2.98) for those treated with antidepressants compared with those given placebo” [(3), p. 431].

In the years that followed, the FDA decision was strongly criticised by some prominent US mental health organisations, psychiatrists and other experts. Some of these critics incorrectly asserted that the FDA warning led to a fall in US youth antidepressant use that resulted in an increase in youth suicide rates:

Using data from rather poorly designed methodology or cherry-picking data from a time series association between antidepressant prescriptions and suicide rates among youth, some have argued that regulatory warnings caused a decrease in antidepressant prescriptions, which then caused more suicides among youth (4).

Most notably, an ecological study by Gibbons et al. incorrectly associated a 22% decrease in selective serotonin reuptake inhibitor (SSRI) prescriptions with a 14% increase in US youth suicide rates between 2003 and 2004, strongly implying causation (5). In fact, in 2004, the year in which suicide rates rose, there was no significant decrease in SSRI use; this did not occur until 2005, when youth suicide rates actually fell. Unfortunately, Gibbons et al.’s study has been very influential:

The claim of higher rates of youth suicide subsequent to the Black Box warning seems to have originated from a 2007 study by Gibbons et al. (5). As of November 26, 2019, Google Scholar indicates this study had been cited 612 times. The Gibbons et al. study’s ﬁnding has thus taken on an air of established truth (4).

Other US experts have expressed concern about the impact of the FDA warning on treatment rates, ignoring the potential of non-pharmacological treatments, and implying that the only options are drugs and no treatment. In a 2007 *New England Journal of Medicine* opinion piece, prominent psychopharmacological experts Richard Friedman and Andrew Leon acknowledged the boxed warning, but declared:

How should physicians deal with the new black-box warning? The real killer in this story is untreated depression, and the possible risk from antidepressant treatment is dwarfed by that from the disease [(6), p. 3].

Outside the US, there has been limited analysis of the long-term impact of the FDA suicidality warning on prescribing or suicide rates. An analysis of trends in SSRI use in the Netherlands and the United Kingdom over 10 years found that UK antidepressant use grew strongly from 2000 to 2010, with somewhat slower growth between 2003 and 2006 (7). In the Netherlands, ‘SSRI use in paediatrics, adolescents, and adults modestly decreased after the first period of media coverage of the warnings, and then recovered’ [(7) p. 4]. The study authors concluded:

changes in SSRI use were temporal, drug-specific and more pronounced in pediatrics and young adults. The twofold increase in SSRI use over one decade indicates that regulatory warnings and media coverage may come and go, but they do not have a significant impact on the overall upward trend of SSRI use as a class in both countries. [(7), p. 1]

This paper discusses how this controversy has played out in Australia, including the role of the Australian Government’s medical product safety regulator, the Therapeutic Goods Administration (TGA), and influential Australian mental health non-government organisations (NGOs) and psychiatrists. Then the paper examines trends in antidepressant use and youth suicide and self-harm, to analyse how competing perspectives on the safety of antidepressant use by young people fit with real-world Australian evidence.

### Backlash: Australian Experts Contest the FDA Warning

In 2005, in response to the FDA’s black box warning, the TGA did not issue the equivalent boxed warning, but instead required the rewording of Product Information and Consumer Information leaflets made available to doctors and consumers (8). This light regulatory approach appears to have been typical of the TGA at that time. From January to September 2005, the FDA issued twenty black box warnings for prescription drugs that were sold in both the US and Australia, but the TGA issued equivalent warnings for only five of them (9).

Subsequent to the FDA’s black box warning and the TGA’s lower-level warnings, several prominent Australian mental health advocacy organisations and influential Australian psychiatrists disputed the antidepressant-youth suicidality nexus, and claimed that the use of antidepressants, on balance, reduced the risk of youth suicide. Some key examples of this are discussed below.

#### Suicide Prevention Australia

Suicide Prevention Australia (SPA) is the “national peak body for the suicide prevention sector” (10). It has received substantial funding from successive Australian Governments to provide guidance on suicide prevention. In 2010, it published a *Youth Suicide Prevention* position statement, which concluded that, “balanced against the risk of not treating youth depression, SSRIs offer some potential to reduce youth suicide” [(11), p. 17].

SPA’s position statement cited Gould et al.’s (2003) literature review (12), spanning 1992 to 2002, to support its positive risk assessment, claiming that it had “shown [SSRIs] to be an effective treatment for youth depression and suicidality”. In fact, Gould et al. had only said it was “plausible” that increased antidepressant prescribing might have decreased youth suicide rates [(12), p. 388].

SPA’s position statement also stated that “the decreased use of SSRIs in Australia has recently been linked to increased youth suicides” [(11), p. 17], but identified no evidence of an Australian decrease in use of SSRIs and no basis for the alleged link with increased youth suicide.

Furthermore, the next sentence in the SPA position statement cited a Cochrane review (13) of SSRI antidepressant use by children and adolescents with depression as supporting fluoxetine as the “most effective SSRI” [(11), p. 17]. However, the Cochrane review reported that “the reduction seen in symptoms was modest” and the limited evidence came from trials of “young people not representative of those presenting for treatment in clinics” [(13), p. 29]. Furthermore, the SPA position statement did not report that the Cochrane review found “an increased risk of suicidal ideation and behaviour for those prescribed SSRIs (RR 1.80, 95% CI 1.19 to 2.72)” [(13), p. 2] and higher rates of adverse events among children and adolescents prescribed SSRIs compared with placebo.

In summary, Australia’s peak body on suicide prevention made unreferenced claims, misrepresented the findings of Gould et al.’s review, ignored key findings of the Cochrane review, and minimised the importance of the FDA and TGA suicidality warnings. In March 2019, nearly a decade after publication, SPA removed the position statement from its website; however, it is still available on the Northern Territory Parliamentary website (14).

#### Orygen Youth Health Research Centre and Headspace National Youth Mental Health Foundation

SPA’s position statement also cited a 2009 *Evidence Summary: Using SSRI Antidepressants to Treat Depression in Young People: What are the Issues and What is the Evidence?* (15), produced by influential Australian mental health non-government organisations, *Orygen Youth Health Research Centre* and *headspace National Youth Mental Health Foundation. Orygen* runs a clinical service in Melbourne and focusses heavily on research (16). The establishment of both *Orygen* and *headspace*—which runs more than 100 mental health clinics across Australia targeting 12 to 25 year-olds—was driven by Professor Patrick McGorry, a very prominent psychiatrist who was 2010 Australian of the Year (17), and was funded primarily by government. McGorry is listed as a clinical adviser on the Evidence Summary, which noted that no antidepressant was approved for use by under-18 year-olds for the treatment of depression, and acknowledged the FDA warning. Overall, the Evidence Summary presented accurate and comprehensive information; however, it concluded that there were “even greater risks of not treating depression with any type of intervention (e.g. pharmacological or psychological intervention)” [(15) p. 2]. This statement is problematic because it groups together two types of treatments with very different harm-benefit profiles, and it provides no evidence for either type of treatment in ameliorating risks associated with depression.

The 2009 Evidence Summary (15) [and the updated 2015 version (18)] stated that, of all SSRI antidepressants, fluoxetine is superior, but even it is only “modestly effective for reducing symptoms of depression in young people” [(15), p. 2]. It recommended that SSRIs may be used to treat moderate to severe depression “within the context of comprehensive management of the patient, which includes regular careful monitoring for the emergence of suicidal ideation or behaviour” [(15) p. 2].

However, an earlier audit of prescribing practices at *Orygen’s* own clinic in 2007 highlighted that “the majority of young people (74.5%) were prescribed an antidepressant before an adequate trial of psychotherapy was undertaken and that less than 50% were monitored for depression symptom improvement and antidepressant treatment emergent suicide related behaviours (35% and 30% respectively)” [(19), p. 1]. There is no publicly available evidence of subsequent prescribing audits, so it is unclear whether *Orygen* has improved its practices since 2007.

It is also notable that in 2009, the same year that *Orygen* produced the original Evidence Summary, it republished an information sheet—*Medications for Depression* (20)—that made no reference to suicidality risks, and contradicted the Evidence Summary by asserting that: “Antidepressants also work well for less severe types of depression” [(20) p. 1]. *Medications for Depression* was still available on *Orygen’s* website on 6 April 2020.

#### Key Opinion Leaders

Patrick McGorry and Ian Hickie, another high-profile professor of psychiatry, have been Australia’s two most influential mental health policy entrepreneurs in the 21st century and have received significant recognition and plaudits for making mental health a prominent issue on the national political agenda (17).

Both McGorry and Hickie have been very prominent in the media, and they continue to have unusually strong influence on the mental health policy and suicide prevention initiatives of Australian state and commonwealth governments of both the Centre-Right (Liberal/National), and Centre-Left (Labor). For example, in June 2019, shortly after the re-election of the Morrison Liberal/National Government, Prime Minister Scott Morrison told the first meeting of his new Cabinet that his government would be the “curse breakers” of youth suicide (21). The first action taken was that Minister of Health Greg Hunt convened an urgent roundtable of a handful of leading mental health and suicide experts that included McGorry and Hickie.

Similarly, when Premier Daniel Andrews’ Victorian State Labor Government established a Royal Commission into Victoria’s Mental Health System in 2019, it appointed McGorry as chairperson of an eight-member Expert Advisory Committee [(22) p.11]. The priority mentioned in the Commission’s terms of reference is to inquire into “how to most effectively prevent mental illness and suicide” [(22) p.592].

In 2000, Ian Hickie was appointed as the inaugural CEO of *beyondblue: the national depression initiative*, a prominent mental health organisation funded by the Commonwealth and state governments. He rapidly developed a high profile in the media, and he was appointed to numerous government and NGO committees, boards, and working groups. In 2006, he was named by the *Australian Financial Review* in its list of Australia’s top 10 cultural influencers for his leadership in mental health and depression in particular (23).

Both McGorry and Hickie, and organisations they have led, particularly earlier in their careers, have received financial support from pharmaceutical companies. In 2008 McGorry declared that he had received unrestricted grants from Janssen-Cilag, Eli Lilly, Bristol Myer Squibb, Astra-Zeneca, Pfizer, and Novartis and had acted as a paid consultant or speaker for most of these companies (24). Organisations he has led, including Orygen (25) and the International Early Psychosis Association (26), have also received funding from AstraZeneca, Bristol-Myers Squibb, Eli Lilly, Pfizer, and Janssen-Cilag.

Hickie has led projects supported by drug industry partners (including Wyeth, Eli Lily, Servier, Pfizer, AstraZeneca) for the identification and management of depression and anxiety. He has served on advisory boards convened by the pharmaceutical industry in relation to specific antidepressants, including nefazodone, duloxetine and desvenlafaxine, and has participated in a multicentre clinical trial of agomelatine effects on sleep architecture in depression. He has also received support for travel to international and national research meetings from the pharmaceutical industry (including Servier, Pfizer, Eli Lilly, Wyeth and Astra Zeneca) (27).

In 2003, the year before the FDA issued its warning, Hickie was a co-author of a frequently cited ecological study (Hall et al., 2003), supporting the use of SSRI antidepressants (28). The abstract concluded:

Changes in suicide rates and exposure to antidepressants in Australia for 1991-2000 are significantly associated. This effect is most apparent in older age groups, in which rates of suicide decreased substantially in association with exposure to antidepressants. The increase in antidepressant prescribing may be a proxy marker for improved overall management of depression. If so, increased prescribing of selective serotonin reuptake inhibitors in general practice may have produced a quantifiable benefit in population mental health. [(28), p. 1]

That study has been cited by Hickie as evidence that increasing antidepressant use rates are likely to be associated with better treatment and fewer suicides (29, 30). However, its results do not support this claim. **Tables 1** and **2** below present the figures from Hall et al. (28) Tables 1 and 2, along with percentage increases.

**Table 1:** Suicide rates (per 100,000) in Australia by sex and age, 1986-2000.

Age group (years)	Men				Women			
	1986-1990	1991-1995	1996-2000	% change	1986-1990	1991-1995	1996-2000	% change
15-24	24.83	26.05	24.81	0%	4.75	5.35	5.74	21%
25-34	28.90	30.32	35.73	24%	6.69	6.60	7.52	12%
35-44	25.04	26.17	30.49	22%	7.01	7.10	8.34	19%
45-54	24.09	24.77	23.78	-1%	8.31	7.17	6.94	-16%
55-64	25.13	22.75	20.68	-18%	8.07	7.04	6.00	-26%
65-74	27.32	23.24	21.91	-20%	8.36	5.95	5.78	-31%
75-84	36.53	30.37	28.68	-21%	8.13	7.63	5.74	-29%
≥85	44.02	40.49	37.45	-15%	6.90	5.53	4.09	-41%

**Table 2:** Estimated use of antidepressants (defined daily dose/1000 people/day) in Australian by sex and age, 1990-2001.

Age group (years)	Men				Women			
	1990-1991	1995	1998-2001	% increase	1990-1991	1995	1998-2001	% increase
15-24	1.2	2.7	14.3	1092%	3.2	7.9	30.7	859%
25-34	3.5	11.8	26.4	654%	10.4	22.2	58.0	458%
35-44	7.6	15.8	36.4	379%	14.6	29.5	73.1	401%
45-54	10.9	20.0	43.0	294%	24.2	47.2	86.7	258%
55-64	14.5	23.6	44.8	209%	32.9	50.9	87.8	167%
65-74	24.1	29.6	47.5	97%	40.0	60.5	103.6	159%
75-84	29.6	38.2	61.3	107%	45.7	67.6	114.2	150%
≥85	29.0	50.7	74.3	156%	29.6	46.1	93.4	216%

The suicide rate data in Table 1 from Hall et al. were examined in three different time-bands: 1986–1990, 1991–1995 and 1996–2000. Over the period 1986–90 to 1996–2000, the per-capita suicide rate (calculated from Hall et al.’s Table 1, which used data provided by the Australian Bureau of Statistics) rose by approximately 16% for Australians aged 15 to 45. There was a 15% fall in the per-capita suicide rate for Australians aged 45 or older. Combining the age-groups, the per-capita suicide rate for all Australians (aged 15 or older) rose by 3%.

Hall et al.’s Table 2 reported antidepressant use rates by five-year age groupings (no data were available for children aged 14 or younger), in three time-bands, 1990–1991, 1995, and 1998–2001. There were massive increases in antidepressant prescribing to all Australians, particularly younger Australians, from 1990–1991 through to 1998–2001.

Because of the growth in suicides among Australians aged 15 to 44 (who made up 59% of the population of Australia aged 15 or older), the life-years lost to suicide would have risen by significantly more than 3% from 1986–90 to 1996–2000. (Note: Similar patterns in suicide rate changes are evident from 1991–1995 to 1996–2000).

These results, detailed in the body of Hall et al., are inconsistent with the abstract’s positive conclusions about the relationship between antidepressant use and suicide. Like Gibbons et al. (5), Hall et al.'s study has been cited frequently (407 times according to Google Scholar as at 30 April 2020), often as evidence that antidepressant use reduces suicide risks (28).

Unfortunately, discrepancies between abstracts and full papers are common in biomedical literature (31) and can be very misleading (32). This occurred in the influential Treatment for Adolescents with Depression Study (TADS) conducted in the USA (33). A comparison of the abstracts of key TADS papers with the detailed results presented in the papers found that the abstracts did not report the significantly higher rate of suicidal ideation/attempt with fluoxetine compared with placebo. In fact, “None of the seven abstracts from TADS publications mentioned the fact that there were four times more suicidal events with fluoxetine than with placebo during the randomised controlled trial, and that this difference was statistically significant” [(34), p. 89; 39].

In a 2007 *BMJ* debate, Hickie cited Hall et al. (2003) (28) as evidence of an inverse causal effect, writing that “increased treatment of depression *reduces* suicides” [italics added] [(30), p. 1]. In addition, he asserted:

Although there has been much hype and regulatory concern about increased prescribing of the new drugs [SSRIs], there is little hard evidence of harm to a significant number of people. The real harm, as evidenced by the suicide statistics, comes from not receiving a diagnosis or treatment when you have a life threatening condition like depression. [(30) p. 2]

In a similar vein, in a 2010 televised debate, ‘Is Depression Being Over-Diagnosed?’, Hickie described as “absolute total nonsense” the assertion that normal sadness was frequently pathologised, and depression was being over-diagnosed and over-treated with medications (35). He went on to praise growing rates of medication use and psychological treatments. He concluded, “when depression’s been treated, suicide goes down, in this country, and many other countries – well demonstrated”.

McGorry’s appointment as 2010 Australian of the Year dramatically increased his public profile and influence as a key thought leader and advisor to government. In the lead-up to the August 2010 Federal election, he partnered with political activist group *GetUp* and addressed candle-light vigils organised to highlight concerns about youth suicide (36).

That same year, Hickie and McGorry co-authored an opinion piece on youth depression in the *Medical Journal of Australia* that acknowledged the FDA warning of “suicidal ideation” but implied that antidepressant use reduced suicide risk (37). They cited an ecological study by Gibbons et al. (38) as evidence that, following the FDA warning, a fall in antidepressant prescribing “was associated with an increase in suicides in young people” in the US [(37), p. 133]. Unlike Gibbons et al. (5), that ecological study made no such claim. It demonstrated a correlation whereby US counties with higher rates of SSRI prescriptions had lower rates of suicide in children and young adolescents. Figures came from 1996-1998, well before any black box warning or decrease in prescribing.

Hickie and McGorry also claimed: “Previous population-based data have indicated a positive relationship between exposure to antidepressants and reduction in suicides” [(37), p. 133]. They cited Simon et al. (39) as evidence that “most [under-18] suicide attempts occur in the month before treatment and then decline sharply once treatment has commenced” [(37), p. 133], without acknowledging that Simon et al. reported only on adolescents *treated with antidepressants*. As suicide attempts are a common trigger for initiating antidepressant treatment, this sharp decline would be expected. It is not evidence that antidepressants reduce suicide risk.

#### General Practitioners

It would be inaccurate to attribute responsibility for the rise in Australia’s youth antidepressant use rates to a few individual key opinion leaders and organisations, or even the psychiatric profession alone. Most antidepressants are prescribed by general practitioners (GPs). For example, in 2014–15 (the year ending 30 June 2015), GPs prescribed 90.4% of the antidepressants prescribed to Australians of all ages. Psychiatrists were directly responsible for only 6.5% (40). Some of the prescriptions by GPs would be repeat prescriptions for treatments initiated by psychiatrists, so the proportion initiated by GPs would be lower than 90.4%. Nonetheless, GPs clearly play a dominant role in the prescription of antidepressants in Australia.

Evidence that some senior GPs believe that antidepressant use prevents suicide in the general population came from an April 2019 radio interview with the President of the Royal College of General Practitioners, Dr Harry Nespolon (41). He was being interviewed in response to media coverage of revelations that approximately 1 in 8 (over 3 million) Australians were dispensed antidepressants in 2018 and that the vast majority of antidepressant prescribing was done by GPs (42). Nespolon reiterated one of the most inaccurate claims in the depression/suicide discourse: “You’re looking at about 1 in 6 people with untreated depression committing suicide”. This echoes a similar claim by Ian Hickie in 2001, while he was CEO of *beyondblue*, that “people with depression have a one in six chance of being dead by suicide” [(43), p. 14].

This grossly inaccurate but persistent claim is likely to have emanated from the 15% suicide rate found in studies from the 1970s and earlier decades of people with *treated* depression, many of whom had received long-term intensive treatment. Diagnostic criteria for depression were much stricter when these studies were conducted, so these patients are not representative of the broader spectrum of people currently diagnosed with depression (44), pp. 147-155).

In 1998, US researchers used a Washington State insurance database to examine the validity of the 15% suicide rate claim. They found that the suicide risk for those treated with depression was many times lower than the 15% lifetime risk estimate and lowest for patients who were not taking antidepressants:

Risk per 100,000 person-years declined from 224 [0.22%] among patients who received any inpatient psychiatric treatment to 64 [0.06%] among those who received outpatient specialty mental health treatment to 43 [0.04%] among those treated with antidepressant medications in primary care to 0 among those treated in primary care without antidepressants. [(45), p. 155]

#### Australian Government

In 2016, the TGA issued a *Medicines Safety Update* highlighting concerns about antidepressants, particularly SSRIs, and suicidality in children and adolescents (46). It discussed research that found that patients and carers were very often not informed about potential risks of antidepressants, including suicidality risks. The TGA’s own prior light regulatory responses to the issue of suicidality and antidepressant use may have contributed to the low level of consumer and carer awareness.

The TGA, like Hickie, has misinterpreted the Australian evidence from Hall et al.’s (2003) study (28) of the relationship between antidepressant use and suicidality. In 2005, in ‘Suicidality with SSRIs: adults and children’ (8), an article in an *Australian Adverse Drug Reactions Bulletin*, the TGA cited Hall et al. (2003) as evidence that “increased prescribing of antidepressants in Australia during 1991-2000 was associated with decreasing suicide rates” (8, p. 14). As explained above, this was wrong. Even Hall et al. stated (in the body of their article) that “The total suicide rate for Australian men and women did not change between 1991 and 2000 because marked decreases in older men and women were offset by increases in younger adults, especially younger men” [(28), p. 2].

In “Suicidality with SSRIs: adults and children” (8), the TGA identified that there was a decrease in the suicide rate among older Australians, but did not acknowledge the more than offsetting increase in suicide by younger Australians. Although the TGA article recognised that Hall et al. did “not demonstrate a causal relationship” [(8), p. 14], it reinforced the suggestion that increasing SSRI prescribing rates may be “indicative of improved overall management of depression” [(8), p. 14]. The TGA accepted the misleading conclusion in Hall et al.’s abstract, without adequately considering the contradictory information in the body of the paper.

### A Decade On: What Does Australian “Real World” Evidence Tell Us?

There is now nearly a decade of real-world Australian data since the *Orygen/headspace* Evidence Summary (15) and the SPA position statement (11) were published and Hickie asserted that the “real harm, as evidenced by the suicide statistics, comes from not receiving a diagnosis or treatment” [(30), p. 2]. The trends are worrying. Multiple sources, including *Orygen* (47), have identified rising child, adolescent and young adult antidepressant prescribing rates (48) and/or increasing rates of suicide [(49), p. 28] and self-harm (50) by young Australians over the last 10 to 15 years.

#### Suicide Rates

**Figure 1:**
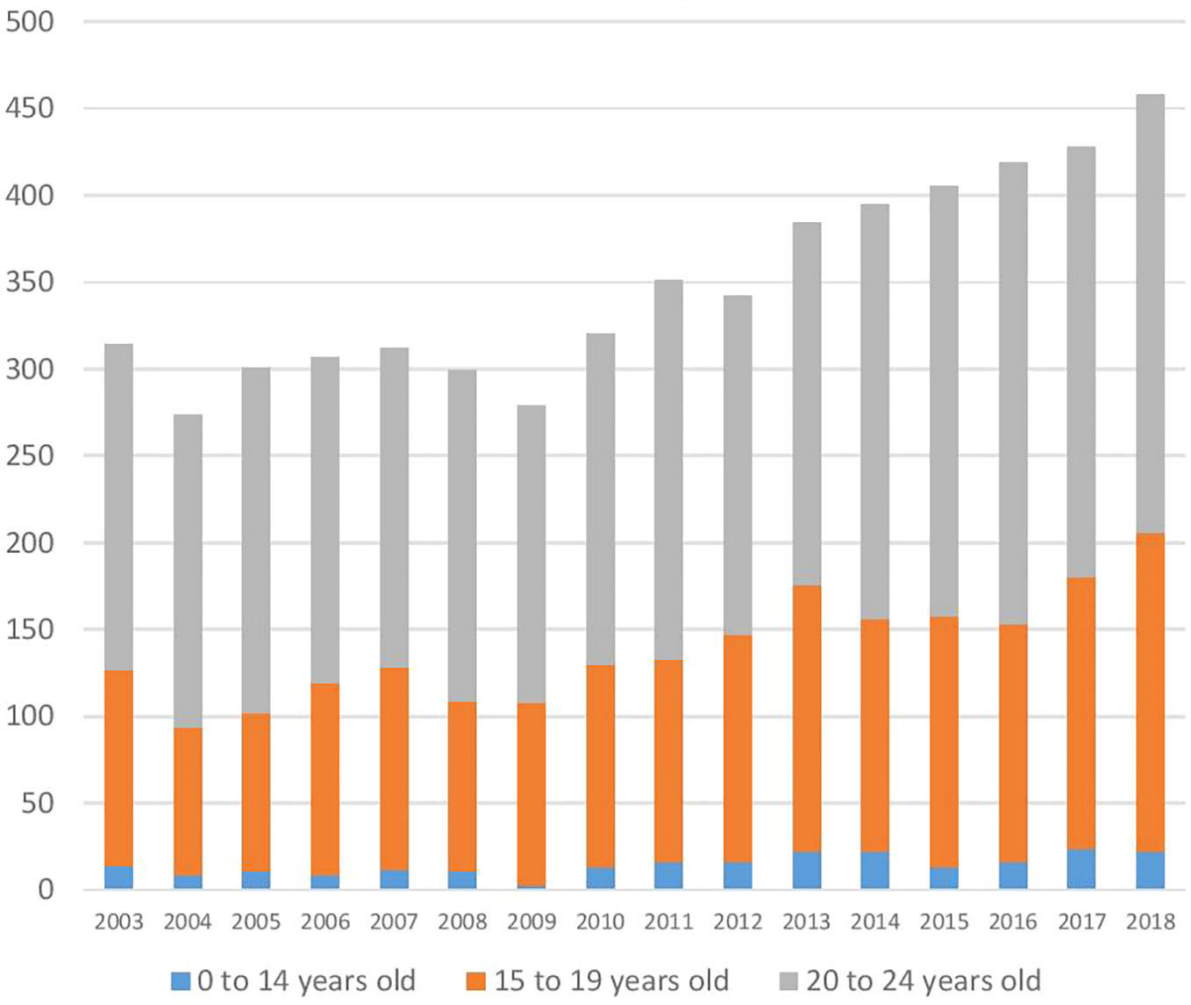
Suicides by young Australians from 2003 to 2018. Source ABS 3303.0 - Causes of Death, Australia, 2018.

Young Australian suicide numbers were volatile between 2003 and 2009; however, since then, there has been a disturbing rise (**Figure 1**). According to the Australian Bureau of Statistics, 279 Australians aged under 25 died by suicide in 2009, and 458 in 2018 (51). This represented an increase of 49.2% from 3.87 to 5.78 deaths by suicide per 100,000 Australians aged 0 to 24 years. Apparent changes in suicide rates must always be interpreted with caution, because of variations in coronial practice over time, but the figures are inconsistent with any drop in suicide in this age group; in fact, they are sufficiently increased to suggest a genuine worsening.

**Figure 2:**
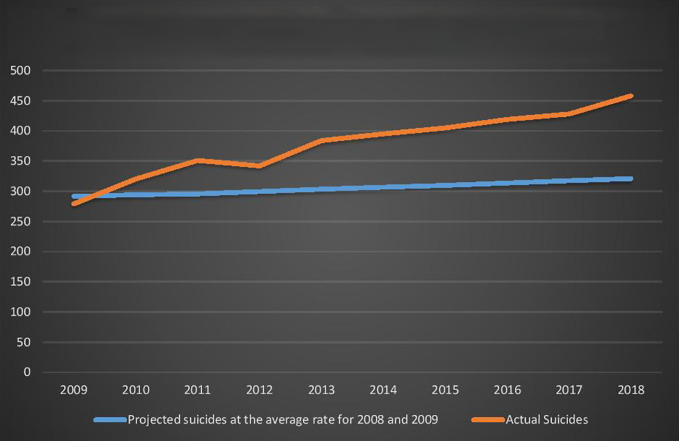
Young Australians (aged 0-24 years) Actual Suicides and Projected Suicides at the 2008 and 2009 average rate.

As demonstrated in **Figure 2** below, if the per-capita rate of under-25-year-old suicide had remained at the average rate for 2008 and 2009 levels, 741 fewer young Australians would have killed themselves between 2010 and 2018.

#### Antidepressant Use

Australians are among the world's biggest consumers of antidepressants. Of 33 OECD countries, Australians were the second highest (behind Iceland) per-capita consumers of antidepressants in both 2000 and 2015 (52). Since 1997/98 SSRI have been the most commonly dispensed antidepressants in Australia. In 1999/2000, they comprised 58% of the number of prescriptions of antidepressants subsidised *via* the Pharmaceutical Benefits Scheme (PBS). In both 2009/10 and in 2018/19, this figure was 45%. The remaining antidepressants—non-selective monoamine reuptake inhibitors (1999/2000 25%; 2009/10 15%; 2018/19 14%), monoamine oxidase inhibitors (1999/2000 5%; 2009/10 1%; 2018/19 1%), and other antidepressants (1999/2000 11%; 2009/10 39%; 2018/19 40%)—were less commonly dispensed (53). Although the relationship between SSRIs and youth suicide appears to have attracted the most scrutiny, the FDA warning applied to all antidepressants (1).

The first *Australian Atlas of Healthcare Variation*, published in 2015, demonstrated there were massive geographical differences in antidepressant and other psychotropic drug use rates, raising concerns about questionable prescribing (54). The *Atlas* also suggested that, for many children, antidepressants are “primarily prescribed for anxiety, rather than depression”, although it also stated that it was not possible to identify the reasons for prescribing. In Australia, no antidepressant is approved for the treatment of depression in people aged under 18, and only two SSRIs (fluvoxamine and sertraline) are approved for children and adolescents with obsessive compulsive disorder (55). Furthermore, there is increasing evidence that antidepressants are not very effective for treating depression in young people (56, 57). Nonetheless, from July 2017 to June 2018, at least 101,174 Australians aged 0 to 17 years (1.8%) were dispensed an antidepressant (58).

In Australia, most commonly used prescription medications, including antidepressants, are subsidised for patients by the Commonwealth (Federal) Government through the PBS, which generates detailed data on dispensing patterns. Analysing shifts in PBS data is the best available method for identifying trends in antidepressant use. **Table 3** below was prepared by the Australian Department of Human Services, using PBS data (58).

**Table 3:** Number of Australians supplied PBS/RPBS prescriptions for antidepressants by age group, 2002–03 to 2017–18.

Patient age group	2002-03	2003-04	2004-05	2005-06	2006-07	2007-08	2008-09	2009-10	2010-11	Break in series*	2011-12	2012-13	2013-14	2014-15	2015-16	2016-17	2017-18
00 to 17 years	44,923	47,677	42,543	36,386	32,578	29,199	30,347	33,730	36,987		50,804	69,973	74,978	79,554	86,348	93,015	101,174
18 to 27 years	145,398	156,252	159,599	146,154	136,065	117,853	121,677	133,358	143,695		194,704	253,607	266,498	283,648	303,605	317,918	328,879
28 to 37 years	238,067	250,464	254,103	235,278	217,999	183,764	184,222	192,473	198,632		274,700	340,775	354,872	368,917	384,064	395,502	406,800
38 to 47 years	285,629	301,409	307,631	289,644	272,274	234,555	236,379	250,893	265,592		374,656	452,103	467,913	479,303	489,379	494,612	498,416
48 to 57 years	283,661	302,182	309,908	295,289	279,378	247,938	249,581	260,861	275,477		390,285	469,699	486,753	498,811	512,352	522,014	533,789
58 to 67 years	207,240	225,779	239,994	252,543	262,648	265,114	279,126	293,293	309,535		379,081	423,334	439,918	451,846	459,805	468,848	482,481
68 to 77 years	186,007	190,029	192,811	196,886	202,847	209,793	220,182	231,139	245,369		265,061	285,244	302,187	317,604	342,563	362,550	383,343
78 to 87 years	134,129	142,598	148,140	155,020	161,565	168,384	175,020	180,777	186,893		194,644	200,953	203,410	206,978	213,394	219,321	227,609
88+ years	31,941	34,714	36,857	39,033	40,728	43,054	47,325	51,310	56,243		61,007	65,078	68,675	71,639	74,664	77,757	80,431
**Total**	**1,556,995**	**1,651,104**	**1,691,586**	**1,646,233**	**1,606,082**	**1,499,654**	**1,543,859**	**1,627,834**	**1,718,423**		**2,184,942**	**2,560,766**	**2,665,204**	**2,758,300**	**2,866,174**	**2,951,537**	**3,042,922**

Includes medicines with an Anatomical Therapeutic Chemical (ATC) classification of N06A - Antidepressants.

Includes patients with PBS and RPBS prescriptions, including under co-payment prescriptions*, supplied from 1 July 2002 to 30 June 2018 and processed on or before 9 January 2019.

Unique patient counts based on PBS data are not available prior to 1 July 2002.

Age of patient based on age at first supply of an antidepressant medicine during the relevant year. Ages grouped by 0-17 (under 18) and then 10 year age groups.

Excludes patients with unknown age.

*Data on under co-payment scripts was collected from 1 April 2012.

**Data prior to 2011-12 does not include under co-payment scripts and is therefore not comparable with data for 2011-12 and later years which includes under co-payment scripts.

2011-12 data includes under co-payment scripts for part of the year (from 1 April 2012). **

PBS online data maintained by the Department of Health and sourced from the Department of Human Services.

Data extracted 10 January 2019.

As stated above, some antidepressant dispensing before April 2012 was not captured, because the cost of some antidepressants did not reach the price threshold for PBS subsidy and therefore those prescriptions were not counted. Since April 2012, data on antidepressant dispensing has included these low-cost antidepressants. Therefore the data since April 2012 are more comprehensive.

PBS data shows that the number and proportion of young Australians (aged 0-27 years) receiving subsidised antidepressants fell significantly after the FDA issued its warning. From the year ending 30 June 2004 (2003-04) to the year ending 30 June 2008 (2007-08), the number and proportion of Australians aged under 28 years receiving PBS subsidised antidepressants fell from 203,929 (2.7%) to 147,052 (1.8%), a 32% decrease.

The Australian Government provided for the period 2003–04 to 2010–11 were collected on a different basis from the data for the period 2012–13 to 2017–18. The data from 2011–12 are a hybrid of both methods. Because of these changes, care needs to be taken interpreting the data.

Despite this limitation, there is a clear trend of increasing dispensing after Australia's depression experts offered their contradictory advice. Following the 2007–08 low point in dispensing rates for young Australians (aged 0–27), the rates rose marginally (1%) in 2008–09, but have increased rapidly thereafter.

- From 2008–9 until 2010–11, there was there was a 17% increase in the likelihood of a young Australian being dispensed one or more PBS subsidised antidepressant.
- From 2012–13 until 2017–18, there was there was a 25% increase in the likelihood of a young Australian being dispensed one or more PBS subsidised antidepressants.
- In 2017–18 approximately 1.8% of Australian children (aged 0 to 17 years) and 9.4% of young adults (aged 18 to 27 years) were dispensed one or more PBS subsidised antidepressant.

As a result of the change in method in 2011–12, it is not possible to calculate the exact per-capita growth in antidepressant use by young Australians since the upswing began in 2008–09. However, assuming the growth rate between July 2011 and June 2013 was the same as average growth rate (6.5%) for the two years before (2009/10 10.8% and 2010/11 7.6%) and after this period (2013/14 4.4% and 2014/15 5.2%), we estimate that, over the decade from 2008–09 to 2017–18, the proportion of Australians aged 0 to 27 years using antidepressants grew from 2.9% to 4.8%.

**Figure 3:**
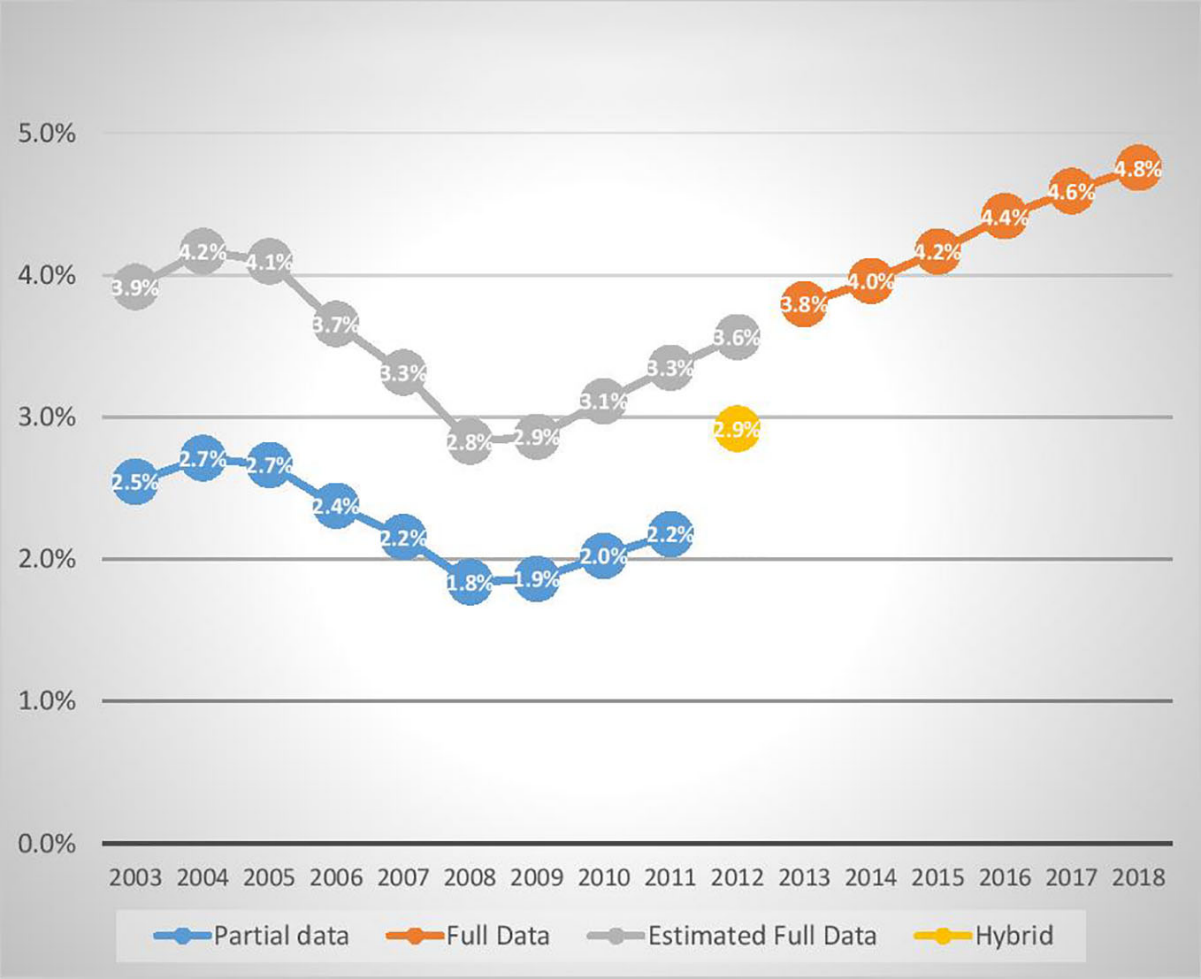
Percentage of young Australians (aged <28) dispensed an antidepressant in the years ended 30 June 2003 to 2018.

**Figure 3** below shows per-capita antidepressant dispensing rates for the 2003 to 2018 financial years (from 1 July to 30 June). The Full Data line (from 2013 to 2018) includes low-cost (unsubsidised) medicines. The Partial Data line (from 2003 to 2011) does *not* include low-cost (unsubsidised) medicines. The Estimated Full Data line (from 2003 to 2011) shows our estimations of the dispensing rates if low cost (unsubsidised) medicines had been included, based on the assumptions stated above. The Hybrid Data point for 2012 reflects the fact that data for that financial year were collected on a different (hybrid) basis.

#### Summary of Antidepressant Dispensing and Suicide Data

Bearing in mind the limitations arising from the changing data collection basis, **Figure 3** demonstrates a pattern of falling dispensing rates from 2004 to 2008, followed by a rebound beginning in 2009. Between 2003–04 and 2008–09, the proportion of Australians (aged 0 to 27) receiving PBS subsidised antidepressants fell by 31%, with most of the fall occurring between July 2005 and June 2008. During the period 2003 to 2009, under-25-year-olds’ per-capita suicide rates were more volatile, with a weak but inconsistent downward trend.

Combining ABS suicide statistics (59) and population data shows that the three-year average of the 0-24 year-old suicide rate for January 2003 to December 2005 was 5% higher than for the period January 2007 to December 2009. The estimated increase in antidepressant prescribing from 2008–09 (2.9%) to 2017–2018 (4.8%) indicates that the probability of an Australian aged 0–27 years being prescribed antidepressants increased by an estimated 66%. Over a similar period (2009 to 2018), the per-capita suicide rate for Australians (aged 0–24) increased by 49%.

In summary, there was a large fall (-31% actual) in dispensing rates for young Australians in the wake of the FDA warning (until June 2008), with no significant change in suicide rates. In contrast, following the contrary advice from Australian experts, there was a large increase in both young Australian suicide (+49% actual) and antidepressant dispensing (+66% estimated) rates.

**Figure 4:**
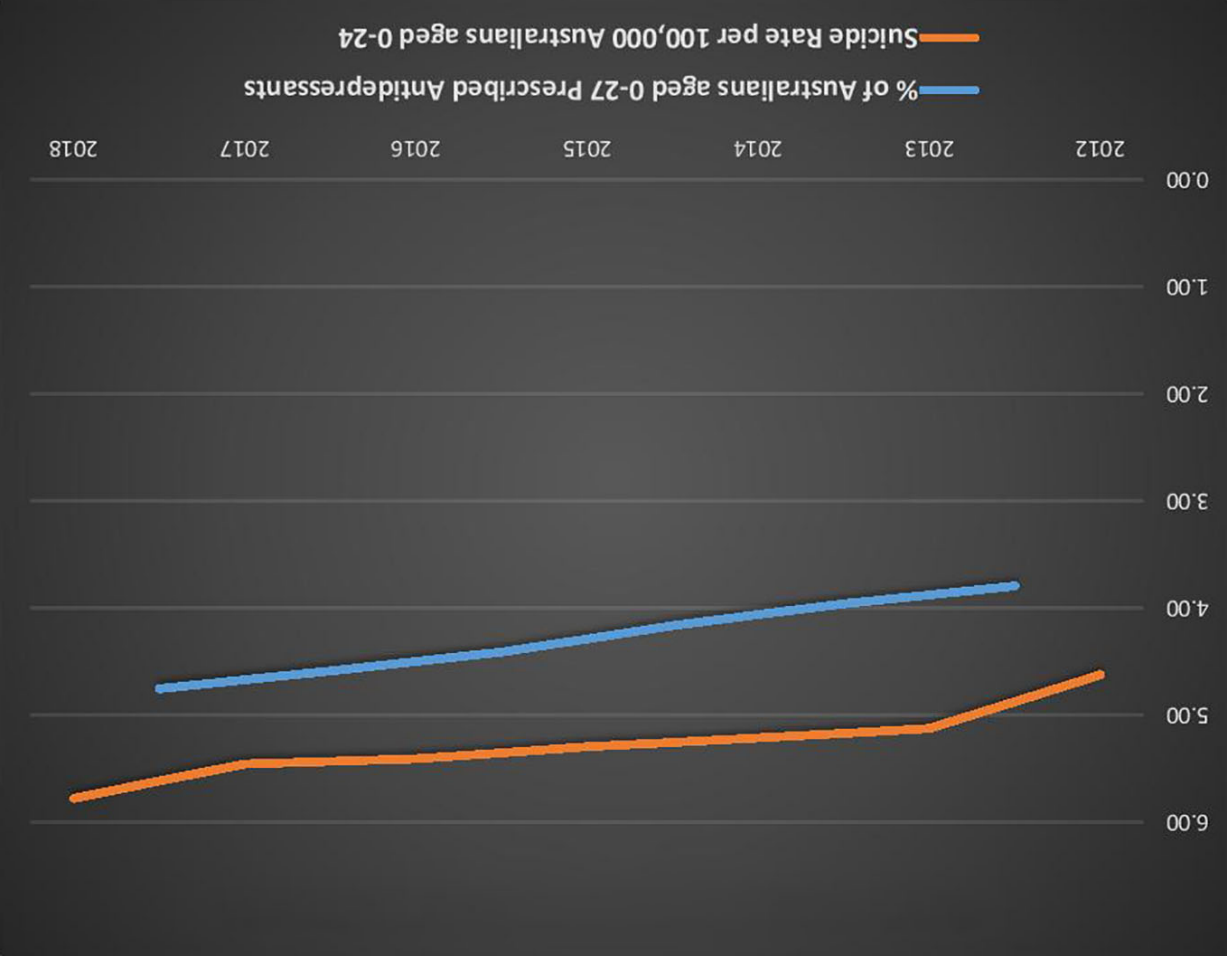
Antidepressant use and suicide rates for young Australians from 2012 to 2018.

These figures need to be interpreted with caution because of the assumptions made about the change in antidepressant dispensing between 2010/11 and 2012/13. However, **Figure 4** below demonstrates that, since PBS antidepressant age related dispensing statistics were first collected in 2012/13 (for the period July 2012 to June 2013), there has been a consistent pattern of rising dispensing rates being associated with rising suicide rates. During this period, the proportion of 0–17 year-olds dispensed an antidepressant rose from 1.3% (in 2012/33) to 1.8% (in 2017/18), and the proportion of 18–27 year-olds increased from 7.7% to 9.4% (58).

Many factors are likely to impact suicide and self-harm rates, and correlation does not prove causation. However, the evidence of increasing young Australian antidepressant dispensing and suicide rates (from 2008–09 onwards) suggests that the advice contradicting the FDA warning may have contributed to increasing antidepressant use, and thereby possibly inadvertently contributed to rising suicide rates among young Australians.

Key Australian mental health organisations and experts have hypothesised alternative explanations for rising youth suicide rates. For example, in 2016, *Orygen*, in collaboration with eleven other organisations (including *beyondblue*, the *Black Dog Institute* and *headspace*), produced a report titled *Raising the bar for youth suicide prevention* (47). The report unambiguously identified that youth and child suicide and self-harm rates were rising, stating:

Over the past 10 years, rather than making inroads into reducing the number of young lives lost to suicide in Australia, there have instead been small but gradual increases in suicide rates … This has mirrored high rates of self-harm among young people. [(47), p. 7]

The report suggested multiple possible causes (e.g., increased use of social media, homophobia and untreated mental illness). In the entire 57-page report, the word ‘medication’ was mentioned once, and antidepressants and SSRIs were not mentioned at all. The possibility that, in line with the FDA warning, rising antidepressant use rates are at least in part responsible for rising youth and child suicide rates, was not discussed.

#### Self-Harm

There is also evidence that antidepressants are commonly used in self-poisoning (overdose). Cairns et al. (2019) found “a concerning increase in child/adolescent [aged 5 to 19 years] self-poisoning in Australia” [(50), p. 6] that corresponded to an increase in psychotropic use rates, particularly SSRI antidepressants. They also found that there was “substantial overlap between the most dispensed psychotropics and medicines most commonly used in self-poisoning episodes” [(50), p. 4].

Cairns et al. presented evidence of increasing antidepressant prescribing rates from 2009 to 2016 in two time-frames. They cited prior research (Karanges et al., 2014) (48) showing that, from 2009 to 2012, antidepressant use by Australians under the age of 25 increased by 25%, and among this group grew fastest in children aged 10–14 (35.5%). Cairns et al. then found that, from July 2012 to June 2016, the number of individuals dispensed SSRIs increased 40% and 35% in those aged 5–14 and 15–19, respectively [(50), p. 1].

Cairns et al. also reviewed data from 2006 to 2016 for New South Wales and Victorian self-poisonings. They found, in the under-20 age group, an increase in intentional annual poisonings of 98% from 2006 to 2016, with most of the growth occurring after 2011. Over the same period, they found a much lower “overall increase of 15% in self-poisoning in persons aged 20 years and over” [(50), p. 3].

These results are consistent with the hypothesis that antidepressants increase the risk of suicidality and self-harm in young people. Furthermore, they provide compelling evidence that the antidepressants prescribed to children and adolescents are frequently the means of self-harm.

In a 2019 article in the *Guardian Australia*, McGorry speculated that a number of possible factors may have contributed to the rising self-harm rates, including “the impact of smartphones, online bullying, and a lack of meaningful face-to-face relationships” and young people’s concerns about “climate change, the casualisation of the workforce, Hecs [university] debt, financial pressures, and social and environmental changes” (60). In addition to identifying these possible contributing factors, McGorry argued that the inability of *headspace* to meet growing demand was “part of the reason why we are seeing increases in self-harm and suicidal behaviour” (60). So, along with other factors, McGorry attributed increasing self-harm and suicidal behaviour to under-funding of headspace, without acknowledging the possibility that the off-label prescribing of antidepressants to under-18 year-olds promoted in the Evidence Summary prepared by *headspace* and *Orygen* might be part of the problem.

Both McGorry and Hickie have recently restated their belief that antidepressants on balance reduce youth suicide. On 24 June 2019, *The West Australian*, published a series of articles including a front page article about a seven-year-old girl who became suicidal on a cocktail of psychotropic drugs including antidepressants. One of the articles, ‘It’s time to rethink kids pills’, highlighted the simultaneous rise in antidepressant prescribing to, and suicide by, young Australians over the last decade (61).

McGorry said that the association made between increased antidepressant use and suicide rates simply did “not hold up … Antidepressants don’t increase suicide. Evidence shows there can be a temporary increase in suicidal ideation… (but) they reduce suicide risk in most”. Hickie was also quoted in the *West Australian* article: “They (critics) are acting like there’s something wrong with increasing treatment … As treatment goes up, we have to be careful, we do run the danger as we increase access … that the trade-off is low-quality care. But what’s the alternative? No care?” Given that Professors McGorry and Hickie continue to be so influential, as evidenced by their prominent role in the post-election suicide roundtable, their continued advocacy of antidepressant use as a means of reducing suicide is significant.

## Conclusion

The Australian pattern of a temporary fall (2004 to 2008) in antidepressant dispensing rates to young people after the FDA warning, followed by a rapid rebound and upswing (2009–2018), mirrors the experience in the Netherlands and the UK. As detailed above in Australia, it is likely that a contributing factor to the rebound and upswing has been the advice of prominent mental health organisations and key opinion leaders.

There have been numerous examples of these influential organisations and individuals incorrectly interpreting or reporting evidence, resulting in inaccurate claims that antidepressant use has been associated with decreased risk of youth suicide. On other occasions in both Australia and the US, prescribing antidepressants or not treating depression has been set up as a false dichotomy, with no consideration of psychosocial treatment options.

Irrespective of the causes, over the last decade, Australian doctors have treated increasing numbers of children, adolescents and young adults with antidepressants. Coinciding with this significant increase in youth per-capita antidepressant dispensing (estimated +66% for those aged 0 to 27 years), there has been an alarming 49% increase in youth per-capita suicide rates (for those aged 0 to 24 years).

Of course, correlation does not prove causation, and many factors impact suicide rates. However, given that the FDA warned that antidepressants were associated with an approximately doubled risk of suicidality relative to placebo, we are not surprised that rising dispensing rates have been accompanied by increasing youth suicide rates.

In the Australian debate following the FDA warning, there were isolated voices, including the three authors of this paper, two as researchers (62) and one as a parliamentarian (63), concerned about the effect of increasing antidepressant use on youth suicide rates. Yet these voices had little impact and, Australia-wide, there appears to have been a culture of uncritical group-think about the relationship between treatment and youth suicide.

The dominant message in the public discourse has been that depression is very common and is serious but easily treated if only troubled young people would seek help (64). Many who propagate this message are undoubtedly well-meaning, but the reality for too many young people is that ‘help’ is nothing more than a short consultation with a GP, a script for an antidepressant, and perhaps a few words of caution about possible side-effects.

Causal relationships cannot be established with certainty until there is a vast improvement in post-marketing surveillance (adverse event monitoring). However, there is clear evidence that more young Australians are taking antidepressants, and more young Australians are killing themselves and self-harming, often by intentionally overdosing on the very substances that are supposed to help them.

## Author Contributions

MW and MR wrote the majority of the section in relation to international evidence on the relationship between antidepressant use, suicide, and self-harm. MW, MR, and JJ collaborated in the writing and editing of drafts and the final paper.

## Conflict of Interest

The authors declare that the research was conducted in the absence of any commercial or financial relationships that could be construed as a potential conflict of interest.
